# Chronic stress induces meiotic arrest failure and ovarian reserve decline via the cAMP signaling pathway

**DOI:** 10.3389/fendo.2023.1177061

**Published:** 2023-08-30

**Authors:** Yiwen Jiang, Jing Xu, Chengqiu Tao, Yunying Lin, Xiaoqi Lin, Ke Li, Qiyu Liu, Hexige Saiyin, Shuanggang Hu, Guangxin Yao, Yun Sun, Feng Zhang, Yu Kang, Congjian Xu, Ling Zhang

**Affiliations:** ^1^ Obstetrics and Gynecology Hospital, Fudan University, Shanghai, China; ^2^ Institute of Metabolism and Integrative Biology, Fudan University, Shanghai, China; ^3^ Center for Reproductive Medical, Renji Hospital, School of Medicine, Shanghai Jiaotong University, Shanghai Key Laboratory for Assisted Reproduction and Reproductive Genetics, Shanghai, China; ^4^ School of Life Sciences, Fudan University, Shanghai, China; ^5^ Shanghai Key Laboratory of Female Reproductive Endocrine Related Diseases, Shanghai, China; ^6^ Department of Obstetrics and Gynecology of Shanghai Medical School, Fudan University, Shanghai, China

**Keywords:** cAMP pathways, chronic stress, CUBIC method, meiotic arrest failure, ovarian reserve decline

## Abstract

Chronic stress is suspected to be a causal factor of female subfertility; however, the underlying mechanisms remain unclear. Here, we found that chronic stress inhibited the cyclic adenosine 3′,5′-monophosphate (cAMP) signaling pathway, leading to ovarian reserve decline in mice. A chronic stress model was constructed using restraint stress for 8 weeks. An elongated estrous cycle and a significant increase in the number of atretic follicles were observed in the stress group. We identified a significant increase in meiotic arrest failure (MAF) in oocytes in the stress group, characterized by condensed metaphase chromosomes, assembled spindles, or polar bodies in the oocytes. Whole-mount ovarian reserve estimation at the single-oocyte level using the CUBIC method (clear, unobstructed brain/body imaging cocktails and computational analysis) revealed a significant decrease in quiescent oocytes from 2,261/ovary in the control group to 1,373/ovary in the stress group. The number of growing oocytes also significantly decreased from 220/ovary in the control group to 150/ovary in the stress group. Real-time quantitative polymerase chain reaction (RT-qPCR) analysis of the meiotic arrest maintenance pathways revealed significant downregulation of *Gpr3*, *Nppc*, and *Npr2* in the stress group. These results indicate that blocking cAMP production contributes to MAF and a decline in ovarian reserve. Overall, we present new insights into the mechanisms underlying chronic-stress-induced oocyte loss and potential targets for ovarian reserve preservation.

## Introduction

Insufficient ovarian reserve, termed premature ovarian insufficiency (POI) in humans, results in reproductive problems and affects 1%–2% of women in the general population (1). A study demonstrated that chronic psychosocial stressors are detrimental to the ovarian reserve (2). However, ovarian dysfunction can also increase stress in women. Whether stress could, in turn, reduce ovarian reserves remains to be elucidated.

Long-term restraint stress has been reported to increase the risk of human diseases, including heart disease and ovarian cancer growth (3–5). Recent studies demonstrated that stress can shorten the life span of mice (6). Several studies have demonstrated that chronic stress specifically affects the ovarian function in rodents, including rats and mice (7–11). These studies revealed a strong association between stress and follicular development. However, the pathogenic effects of chronic stress on ovarian function manifest mainly as an inhibition of gonadotropin release. Whether other intraovarian factors are involved in this process remains unclear.

Chronic stress results in the increased production of stress mediators, such as norepinephrine (NE) and epinephrine, via the hypothalamic–pituitary–adrenal (HPA) axis. Under stressful conditions, HPA axis activation can suppress the activity of the hypothalamic–pituitary–gonadal (HPG) axis at multiple levels (12, 13). The HPG axis is mediated by the release of gonadotropin-releasing hormone (GnRH) from the hypothalamus in response to diminished circulating sex hormone levels in women. GnRH stimulates the release of follicle-stimulating hormone (FSH) and luteinizing hormone (LH) to stimulate follicular development and estrogen production, respectively, and causes ovulation (14). The paraventricular nucleus of the hypothalamus (corticotropin-releasing factor), anterior lobe of the pituitary gland (adrenocorticotropic hormone), and adrenal gland (glucocorticoid synthesis and secretion from the zona fasciculata) compose the HPA axis and could also play important roles in the regulation of ovarian function. In response to stress, the adrenal glands secrete glucocorticoids, such as cortisol, to suppress LH release in the pituitary and sex hormone production in the gonads (15). The brain stem’s noradrenergic neurons, sympathetic adrenomedullary circuits, and the parasympathetic system also respond to stress. However, it remains unclear whether long-term mild stress suppresses ovarian function.

Restraint stress is commonly used in animal models to mimic physical and psychological stress in human beings (5, 16, 17). Gao et al. reported that chronic restraint stress in mice impaired oocyte development potential through cumulative effects on growing follicles (11). Xu et al. also reported that chronic restraint stress induced excessive activation of primordial follicles and an increase in primary follicles, but a decline in secondary follicles and the corpus luteum (10). Fu et al. reported that the number of small follicles was significantly reduced after chronic stress in rats, promoting the development of premature ovarian insufficiency (18). These studies have demonstrated that stress can affect follicular development, at least in animal models. However, the effects of stress on small-follicle development remain unclear.

In the above studies, the ovarian reserve was mostly evaluated by counting the follicles on tissue slices, which only provided part of the oocyte reserve in the ovary, and the primordial follicles were very difficult to identify because of their small size. In addition, the same follicle could have been recalculated on several slices, leading to false-positive results in ovarian reserve analysis. A more accurate method is needed to improve ovarian reserve evaluation. Tissue reconstruction via lipid clearing, followed by antibody staining and fluorescence microscopy, is a new method for *in situ* analysis of whole-mount cellular numbers and cell biology. Clear, unobstructed brain/body imaging cocktails and computational analysis (CUBIC) is a well-known tissue clearing and imaging method. It has been used to clear whole organs, including the mouse brain, ovaries, and whole body (19–21). The 3D images revealed a single-cell phenotype within transparent intact tissues.

In this study, we constructed a stress model to analyze the long-term effects of mild chronic stress on the ovarian reserve. Using the CUBIC method, we reconstructed 3D ovarian images that revealed a more accurate landscape of the ovarian reserve than the classic tissue slicing method. We also demonstrated that inhibition of the cyclic adenosine 3′,5′-monophosphate (cAMP) pathway could be a potential mechanism of chronic-stress-induced follicular developmental anomalies.

## Materials and methods

### Animal models

The animal studies were approved by the Institutional Review Board of Fudan University and were carried out in accordance with the recommendations of the Guide for the Care and Use of Laboratory Animals of the US National Institutes of Health. Female C57BL/6 mice were bred in a specific-pathogen-free barrier facility. We used a restraint stress procedure as reported previously (5). Chronic restraint stress was induced by placing the mice in a physically restrained system. We randomly assigned 8-week-old mice to the control (n=20) and stress groups (n=20). In the stress group, the mice were stressed for 4 h/day from 10:00 to 14:00 and then raised in normal cages. Five mice were bred in each normal cage. The mice in the control group were housed in normal cages. All animals were deprived of food and water between 10:00 and 14:00. Tissues and serum were harvested after 8 weeks of stress. Body weights were recorded every 2 weeks. Mice were anesthetized with isoflurane to collect the blood by hepatic portal vein puncture for hormone analysis. The ovaries and uteri were weighed before fixation in paraformaldehyde (PFA, Sangon Biotech, E672002).

### Steroid and gonadotropin assays

Blood samples from the control and stress groups were collected and centrifuged (3,000 rpm, 15 min) to separate the serum after standing for 12 h at 4°C. The collected serum was stored at −80°C until further analysis. The concentrations of steroids and gonadotropin were assayed using an enzyme-linked immunosorbent assay (ELISA) kit according to the manufacturer’s instructions (**Supplementary Table S1**).

### Estrous cycle detection

The estrous cycles of all mice were monitored daily during the first 4 weeks. Vaginal cells were collected by saline washes and analyzed by Wright–Giemsa staining. Estrous cycle stages were defined by predominantly nucleated cells, predominantly cornified epithelial cells, cornified epithelial cells with some nucleated cells and leukocytes, and predominantly leukocytes for the proestrus, estrus, metestrus, and diestrus stages, respectively.

### Tissue slicing for follicle counting

For histological analysis of growing follicles, the right ovaries from the mice were fixed at 4°C for 24 h in 4% PFA before being dehydrated and embedded in paraffin. For microscopic analysis, consecutive series of sections were cut into 5-μm-thick slices. The slices were deparaffinized in xylene and rehydrated using a decreasing ethanol gradient. Afterwards, they were stained with hematoxylin and eosin (H&E). The middle piece from every 30 slices was used per ovary. The slices were scanned using Pannoramic MIDI (3DHISTECH) and viewed using the Case Viewer. Follicles with ≥ 1 layer of cuboidal granulosa cells were treated as growing follicles. The follicles without visible oocytes were treated as follicles. When counting growing oocytes, only those with an apparent nuclei were considered normal meiotic arrest oocytes. Oocytes with condensed chromosomes, apparent spindles, or extruded polar bodies were considered as meiotic arrest failure (MAF) oocytes (22).

### Tissue clearing

The CUBIC method was used in this study to reconstruct 3D ovarian images (19, 23). Reagent-1 included water (35%, weight/weight), urea (25%, w/w), Quadrol (25%, w/w), and Triton-X 100 (15%, w/w). Reagent-2 included water (15%, w/w), urea (25%, w/w), sucrose (50%, w/w), and triethanolamine (10%, w/w) as **Supplementary Table S2**. The ovaries were fixed in ice-cold PFA for 24 h and then transferred to sugar (30%, w/v) for 24 h. After washing in phosphate-buffered saline (PBS) three times, the ovaries were incubated in CUBIC reagent-1 for 5–7 days on a 37°C shaker until the ovaries became transparent. The ovaries were washed in 5 ml of PBS for 2 h. Five or more repetitions of this procedure were required until no bubbles were observed in the PBS. The ovaries were then transferred into new bottles for primary antibodies DEAD box helicase 4 (DDX4, or with tumor protein P63) incubation on a 37°C shaker for 3 days. Information on antibodies is shown in **Supplementary Table S3**. Antibodies were diluted in PBS containing 10% donkey serum and 1% Triton-X 100. The ovaries were then washed five times (1 h/time) in 5 ml PBS. The washed ovaries were transferred to new bottles for secondary antibody incubation on a 37°C shaker for 1 day. Fluorescence secondary antibodies were diluted in PBS containing 5% donkey serum and 0.5% Triton-X 100. Stained ovaries were washed five times in 5 ml PBS (1 h/wash) and re-cleared in reagent-2 before mounting. The stained ovaries were stored in CUBIC reagent-2 for several weeks with minimal fluorescence quenching.

### Fluorescence microscopy

Butyl rubber was used to create U-shaped grooves on the slides (**Supplementary Figure S1A**). One ovary was placed in the groove, which was filled with CUBIC reagent-2. The groove was covered with a piece of cover glass without air bubbles between the ovary and the cover glass. A Nikon A1 confocal microscope was used for the fluorescence imaging. For the 3D reconstruction, each ovary required 2×2 or 3×2 stacks of imaging with a 10% overlap between adjacent stacks (**Supplementary Figure S1B**). In each stack, the Z-series step size was 3 μm.

### Image processing

Imaris Viewer version 9.0.1 was used for the image processing. The oocytes were identified by spot transformation and manual correction. To construct the spot groups, the principal parameters were set and adjusted according to the size of the oocytes, quality of the object (minimum score=100), and intensity of the signal. The “co-localize” algorithm was also applied to distinguish the spot groups. We obtained four groups of spots to indicate different oocyte groups. As shown in **Supplementary Figure S2**, DDX4-positive oocytes had different cell sizes, and enlarged cells indicated a growing state of the oocytes. Group 1 contained the smallest oocytes with a similar cell size, indicating quiescent oocytes. The cell size parameter of group 1 was set to 17 μm. When constructing the group 1 spot, we automatically obtained group 2 spots after colocalization analysis. Group 2 spots indicated wrongly recognized spots and colocalized with the spots from group 4. Therefore, spots from group 2 were ignored when analyzing the oocyte number. Spots in groups 3 and 4, whose cell size parameter was set to 50 μm, identified the growing oocytes with differently enlarged oocyte sizes. The total number of spots in groups 3 and 4 indicated the growing oocyte numbers.

### mRNA expression level analysis

Total RNA and protein were extracted from the left ovaries of the mice in both groups using an AllPrep RNA/Protein Mini Kit (Qiagen, 80404). Total RNAs (1 μg) were immediately converted into cDNAs using PrimeScript RT Master Mix (Perfect Real Time, Takara, RR036A). The cDNAs were diluted to 10 ng for use as templates in the subsequent qPCR with the AceQ qPCR SYBR Green Master Mix (Vazyme). Mouse α-tubulin was used as an internal control. Expressions of the target genes were normalized to tubulin, and mRNA levels were quantified using to the 2^−ΔΔCt^ method. The primers used for the expression analysis are shown in **Supplementary Table S4**.

### Statistical analysis

Data are presented as mean ± standard error of the mean (SEM). An unpaired t-test was used to test the difference in oocyte number between the stress and control groups. Fisher’s exact test was used to determine the significance of the MAF number between the stress and control groups, *p<0.05, **p<0.01, and ***p<0.001.

## Results

### Estimation of chronic stress model

**Figure 1:**
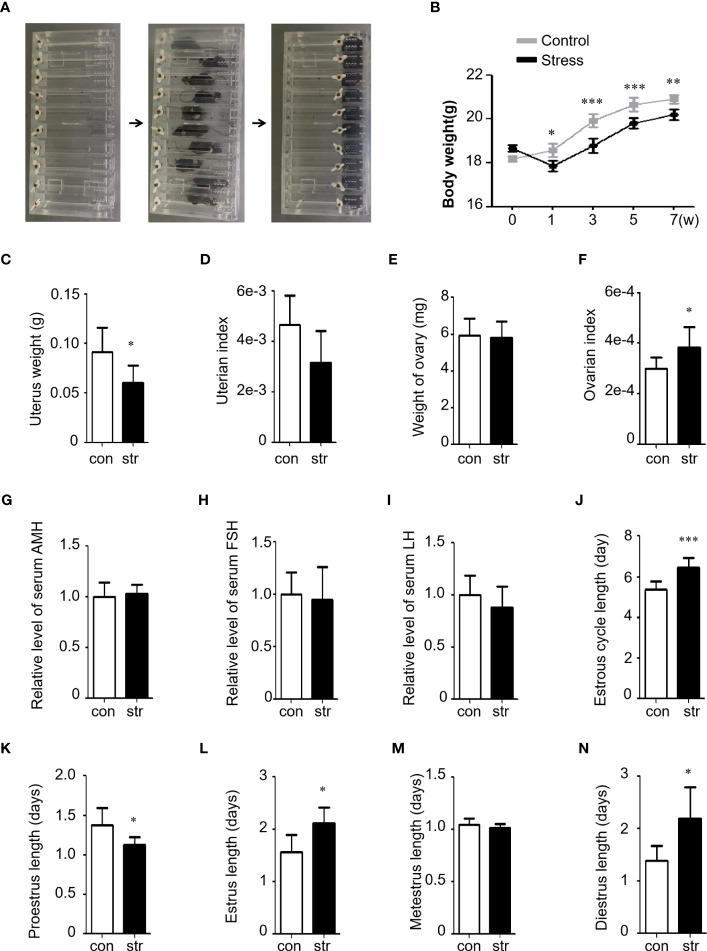
Restraint stress induced ovarian dysfunction in female mice. **(A)** Stress was induced by motional restraint of C57BL/6 female mice in front of the box. **(B)** Body weight of mice in the control (n=10) and stress (n=10) groups. w, week. **(C, D)** Uterus weight and index (tissue weight/body weight) of the mice in the control (n=10) and stress (n=10) groups. **(E, F)** Ovary weight and index (tissue weight/body weight) of the mice in the control (n=10) and stress (n=10) groups. The weight indicates the total weight of the left and right ovary of a mouse. **(G–I)** Serum hormonal levels of AMH **(G)**, FSH **(H)**, and LH **(I)** in the stress group (n=8) relative to the control group (n=8). **(J–N)** Length (days) of the estrus cycle **(J)** and Proestrus **(K)**, Estrus **(L)**, Metestrus **(M)**, and Diestrus **(N)** in the control (n=10) and stress (n=10) groups. con, control; str, stress. *p<0.05; **p<0.01; ***p<0.001. t-test.

A chronic restraint stress model was established with 4 h of daily restraint stress for 8 weeks (**Figure 1A**). A significant decrease in body weight was observed after the first week of stress exposure (**Figure 1B**), which is consistent with previous reports (10). Uterine weight was also evaluated, and we found a significant decrease in the stress group (**Figure 1C**). However, no significant difference was found in the uterine index (tissue weight/body weight) between the groups (**Figure 1D**). Ovarian weight showed no significant change between the groups (**Figure 1E**); however, the ovarian index (tissue weight/body weight) significantly increased in the stress group (**Figure 1F**), similar to a previous study. The serum levels of anti-Müllerian hormone (AMH), luteinizing hormone (LH), and follicle-stimulating hormone (FSH) were also tested (**Figures 1G–I**). The results showed a slight change between the stress and control groups; however, no significant difference was observed. In addition, morphological characterization of the vaginal smears at every stage was performed using Giemsa staining and light microscopy (**Supplementary Figure S3**). Compared to the controls, irregular estrous cycles were observed in the stress group, including shortened proestrus, prolonged estrus, and diestrus (**Figures 1J–N**). Collectively, these results indicate that chronic stress affected ovarian function in mice.

### Chronic-stress-induced oocyte death and meiotic arrest failure

**Figure 2:**
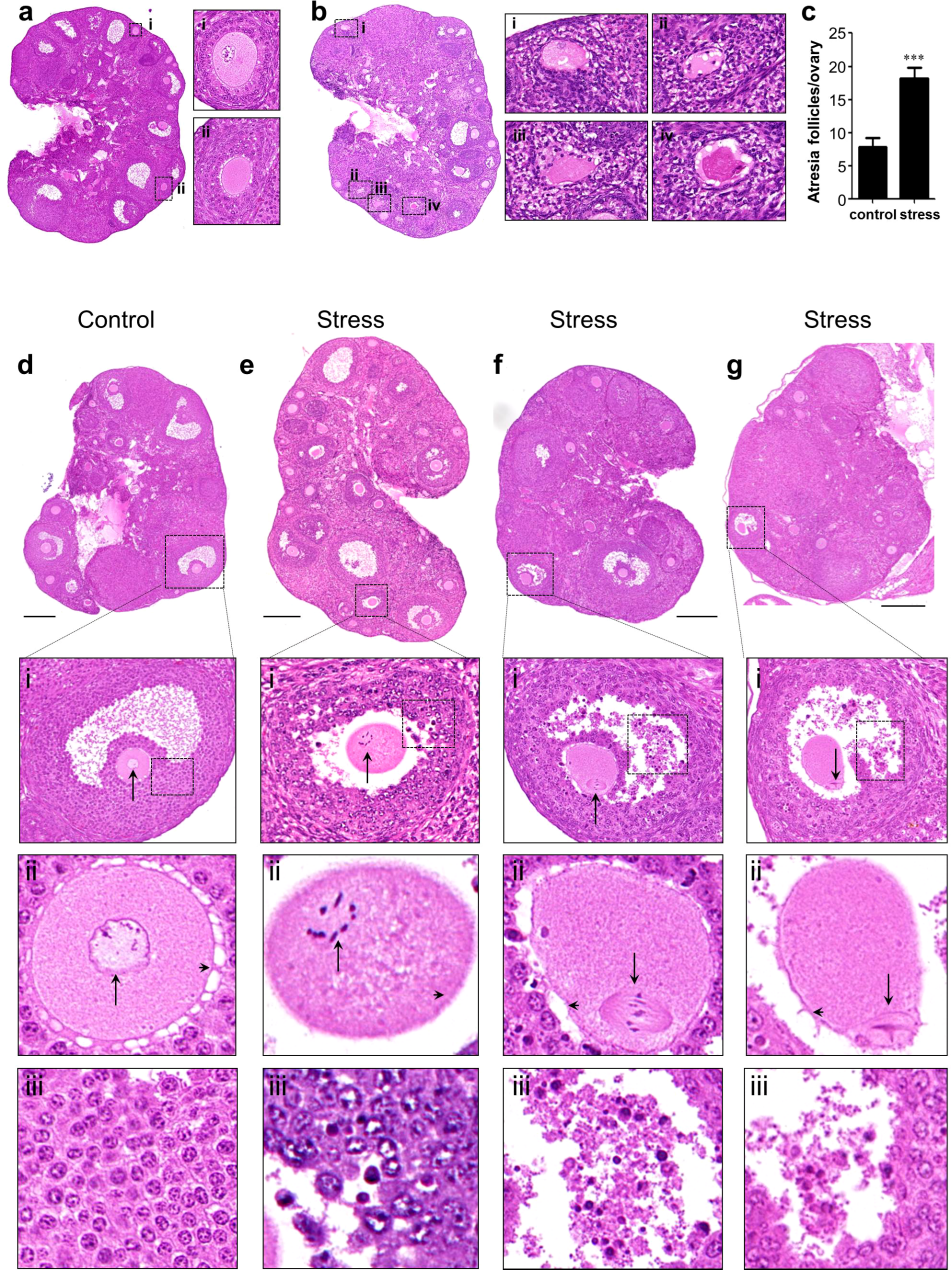
Histological analysis of ovaries. **(A, B)** Atresia follicle analysis in an control ovary **(A)** and a stress ovary **(B)**. Magnified views of boxed areas are shown in adjacent panels and indicate healthy follicles in the control ovary (i–ii) and atresia follicles in the stress ovary (i–iv). Healthy growing follicles have round oocyte and regularly arranged granulosa cells. Atresia follicles manifested with oocyte malformation and irregularly arranged granulosa cells. **(C)** Statistical analysis of atresia follicles/ovary in the control (n=11) and in stress (n=7) groups. **(D–G)** Histological analysis oocytes with meiotic arrest failure (MAF). **(D)** Normal oocytes with a germinal vesicle are shown in magnified views. **(E–G)** MAF oocytes have condensed chromosomes, metaphase spindle, and first polar bodies as indicated by black arrows in the magnified views. Arrowheads indicate normal gap junctions in the control ovaries and broken gap junctions in the stress ovaries (magnified views of row ii). Deciduous granulosa cells were observed in the MAF follicles in row iii. ***p<0.001, t-test. Scale bar: 250 μm.

To analyze the effect of chronic stress on follicle development, we performed serial slicing and hematoxylin and eosin (H&E) staining of ovaries from the control and stress groups. The middle sections of every 30 slices was used for light microscopy and analysis. The total number of follicles or follicles at different developmental stages did not differ significantly (**Supplementary Figures S4A, B**). In addition, no significant differences were detected in the ratio of primary follicles to primordial follicles, number of corpus luteum, and total growing follicles between these groups (**Supplementary Figures S4C–E**). However, we observed a significant increase in atretic follicles in the stress group compared to the control group (**Figures 2A–C**).

To analyze the events leading to oocyte death, we focused on the most important events in meiotic arrest maintenance in growing oocytes. Normally, growing oocytes maintain the germinal vesicle (GV) during development until ovulation (**Figure 2D** and magnified figures). However, we found a noticeable number of MAF oocytes in the stress group, characterized by condensed chromosomes (**Figure 2E** and magnified figures), assembled spindles (**Figure 2F** and magnified figures), or release of the first polar body (**Figure 2G** and magnified figures). We detected nine MAF oocytes out of 249 growing oocytes (3.6%, 9/249) in the control group. However, the frequency of MAF oocytes was significantly increased in the 171 growing oocytes (25/171, 14.6%, 25/171) in the stress group (**Table 1**; Fisher’s exact test, *p*<0.0002).

**Table 1:** Number of MAF oocytes in the control and stress groups.

Group	Number of ovaries	Number of slices	Number of growing oocytes	Number of MAF oocytes	Average number of MAF on each slice	% of MAF in total oocytes
Control	11	33	240	9	0.27	3.6%
Stress	7	21	146	25	1.19	14.6%^a^

a *P*<0.0001, Fisher’s exact test.

We also noticed that most of the gap junctions were broken down in the MAF follicles (**Figures 2E–G** and magnified figures) but remained intact in the control ovary (**Figure 2D** and magnified figures). In addition, granular cells were dropped into the follicular fluid in the stress group (**Figures 2E–G**) but close to the follicle wall in the control group (**Figure 2D**). These results indicate that chronic stress could lead to the breakdown of the junctions between granulosa–granulosa cells and granulosa–oocytes and also disturbs meiotic arrest maintenance in growing follicles, resulting in follicle atresia and oocyte loss.

### Chronic stress led to ovarian reserve loss

**Figure 3:**
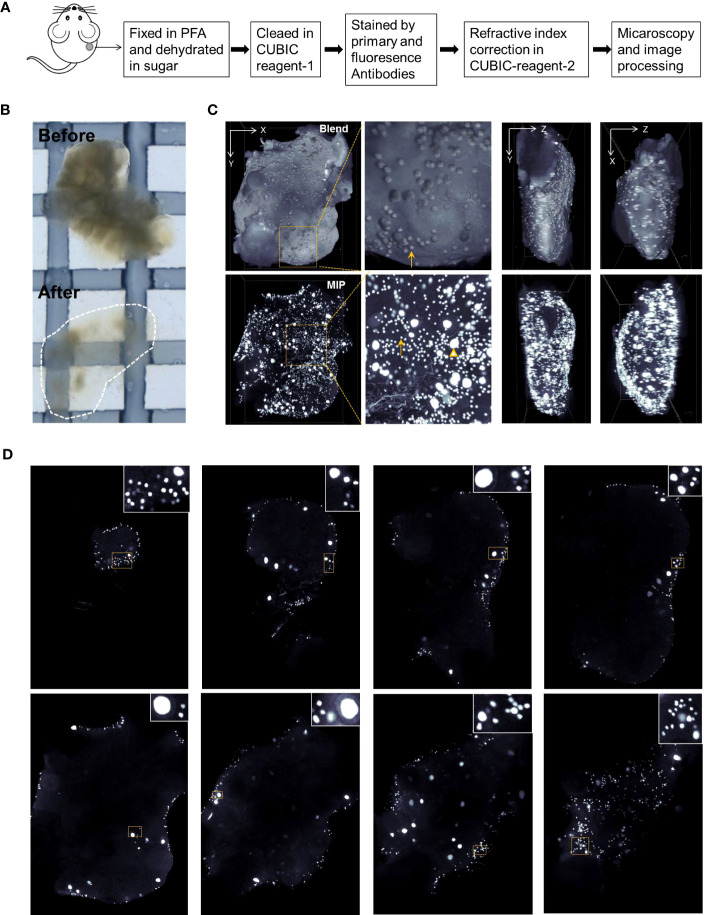
Ovarian reconstruction method. **(A)** Protocol of ovarian reconstruction. **(B)** Ovaries before and after clearing by CUBIC reagent-1 cocktail. The ovaries are immersed in the CUBIC reagent-1 for light microscopy. **(C)** DDX4 antibody and goat anti-rabbit-cy3 fluorescence antibody were used to stain the oocytes specifically. Three-dimensional (3D) views from X–Y, X–Z, and Y–Z directions of the reconstructed ovaries. Blend views provide the surface information of the reconstructed ovaries and Maximum Intensity Projection (MIP) views present the fluorescence signals in the inner space. Magnified views of boxed areas are shown in adjacent panels. Arrows indicate the oocytes from primordial follicles and triangles indicate the oocytes with enlarged cell size from the growing follicles. Also see Supplementary **Figure S5** and S6. **(D)** Digital slices from interspersed layers of reconstructed 3D images show the oocytes distribution.

Ovarian aging is characterized by an accelerated loss of the ovarian reserve, which is usually estimated by ovarian tissue section analysis. Oocytes in primordial follicles are difficult to count accurately based on tissue sections. In this study, we used the established CUBIC tissue-clearing method to reconstruct 3D ovarian images from the stress and control groups (**Figure 3A**). Lipids in the ovaries were cleared using CUBIC reagent 1, and the tissue became transparent (**Figure 3B**).

DDX4 antibodies were used to stain the oocyte cytoplasm. To test DDX4 antibody specificity, ovarian tissue slices were double-stained with fluorescence immunostaining for DDX4 and DAPI, or DDX4-P63. As shown in **Supplementary Figure S5A**, DDX4 was specifically located within oocytes from growing and primordial follicles. Additionally, DDX4 colocalized with another germ cell nucleus-specific marker P63 (**Supplementary Figure S5B**).

After antibody staining, the ovaries were immersed in CUBIC reagent-2 to adjust the reflective index. Fluorescence microscopy was performed as described in the *Materials and methods*. The reconstructed ovarian images were rendered as 3D images using the blending and Maximum Intensity Projection (MIP) modes to view the oocytes on the surface and inner space, respectively (**Figure 3C**; **Supplementary Movie S1**). Clipped views of the 3D images also showed the spatial localization of oocytes in the different imaging layers (**Figure 3D**; **Supplementary Movie S2**).

**Figure 4:**
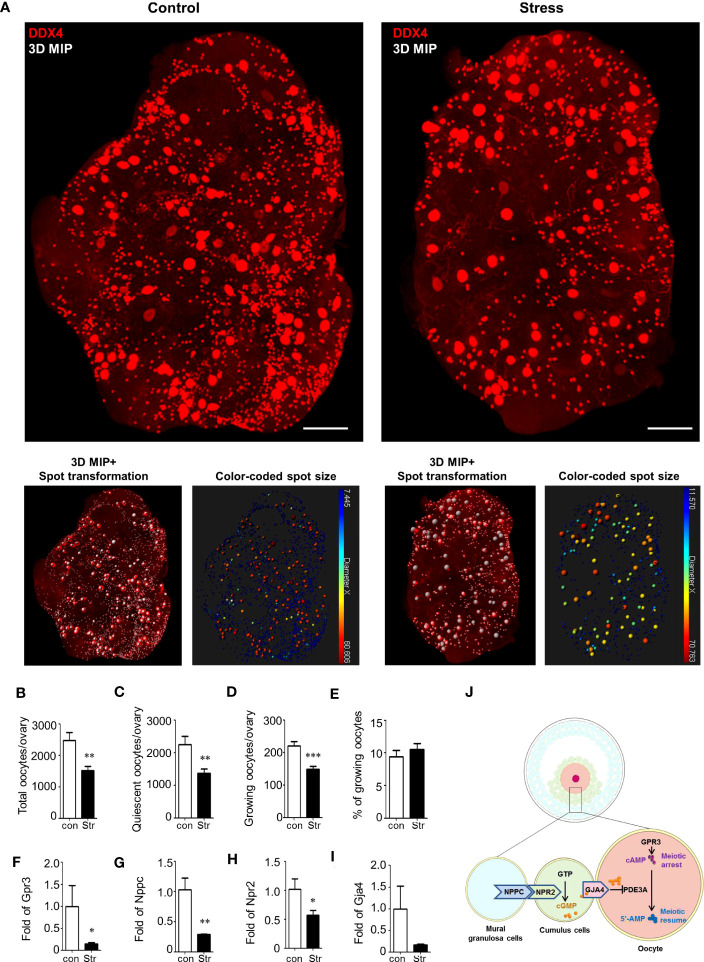
Ovarian reserve analysis by ovarian reconstruction and underlying mechanism study. **(A)** Exemplified reconstructed 3D ovarian images from the control and stress groups. The oocytes are stained by DDX4 antibodies and identified by spot transformation on Imaris software. The spots are coded by colors based on spot sizes. Quiescent oocytes were indicated by blue spots and growing oocytes as green-to-red. **(B–D)** Statistical analysis of the number of total oocytes, quiescent oocytes, growing oocytes, and percentage of growing oocytes in total oocytes per ovary in the control (n=7) and stress (n=12) groups. Mean with SEM are showed for each group. **(F–I)** Relative expression level of *Gpr3*, *Nppc*, *Npr2*, and *Gja4* in the control (n=3) and stress (n=5) groups. **(J)** Schematic diagram of the molecular mechanism underlying stress-induced MAF. First, stress blocks the expression of *Gpr3* and inhibits the synthesis of cAMP in the oocytes. Second, stress blocks the expression level of *Nppc* and *Npr2* and inhibits cGMP synthesis in cumulus cells. Third, stress blocks the *Gja4* to form gap junctions and inhibits the transfer of cGMP, which blocks the activity of *Pde3a*, from cumulus cells to oocytes. Scale bar: 300 μm. *p<0.05; **p<0.01; ***p<0.001.

In the 3D ovarian images from the control and stress groups, the quiescent oocytes had a similar cell size, with a diameter of 12–17 μm, and growing oocytes were between 17 and 60 μm (**Figure 4A**; **Supplementary Figures S6A–C**). Using spot transformation and manual correction, we determined the number of spots indicating the oocyte number in the ovaries. We found a significant difference in the total number of oocytes between the control (2,482 ± 254, mean ± SEM) and stress groups (1,523 ± 137, *p*<0.0022) (**Figure 4B**). The number of quiescent oocytes was also significantly lower in the stress group (1,373 ± 132, *p*<0.0029) than that in the control group (2,261 ± 246; **Figure 4C**). Moreover, we found a significant decrease in the number of growing oocytes in the stress group (150 ± 9, −32%) compared to that in the control group (221 ± 13, *p*<0.0004); however, the ratio of growing oocytes to total oocytes did not significantly differ (**Figures 4D, E**). These results indicate that accurate oocyte quantification based on the CUBIC tissue reconstruction method is applicable in ovarian reserve analysis and that chronic stress could lead to accelerated oocyte loss and ovarian reserve decline, even though we did not observe follicle number changes based on the tissue slicing method.

### Chronic stress inhibited the cAMP signaling pathway in the ovary

To reveal the potential reasons for MAF, we measured the expression levels of genes involved in meiosis arrest-maintaining pathways using RT-qPCR. As shown schematically in **Figure 4J**, meiotic arrest was maintained by high levels of cAMP in oocytes. cAMP can be generated in oocytes or transferred from cumulus cells to the oocytes. cAMP is produced under the regulation of G-protein-coupled receptor 3 (*Gpr3*) in oocytes (24–26). Significant downregulation of *Gpr3* was detected in the stress group (**Figure 4F**). cAMP in oocytes could also be hydrolyzed by the oocyte-specific phosphodiesterase *Pde3a (* 27). However, *Pde3a* expression was not significantly affected by stress (**Supplementary Figure S7A**).

Considering that a high level of cGMP in oocytes is also necessary to inhibit the activation of PDE3A and prevent the hydrolysis of cAMP, we measured the pathways involved in cGMP synthesis and transport. cGMP is produced in granulosa cells under the promotion of natriuretic peptide C (*Nppc*) and natriuretic peptide receptor 2 (*Npr2*) and is transferred into oocytes via gap junctions (22). *Nppc* and *Npr2* levels showed a significant decline in the stress group (**Figures 4G, H**). Gap-junction protein 4 (*Gja4*) expression also showed a decreasing trend in the stress group (**Figure 4I**). To explore the potential mechanism leading to the downregulation of *Nppc*, we assessed the expression level of an upstream gene, ring finger protein 36 (*Zfp36*), which is mainly expressed in mural granulosa cells to degrade the mRNAs of *Nppc* (28). There were no significant differences between the control and stress groups (**Supplementary Figure S7B**). Similarly, the signaling pathway *Lhcgr*/*Mapk1*/*Elk1*/*Egr1*, which is an upstream pathway that regulates the expression of *Zfp36*, also showed no significant change in the stress group compared to the control group (**Supplementary Figures S7C–F**). These results suggest that other unknown pathways regulate the expression of *Nppc* in the ovaries.

To test other potential underlying mechanisms that could predict oocyte survival, we also measured the expression levels of the oocyte-specific growth factor *Gdf9* and pellucida zone protein *Zp3*; however, no significant differences were found between the groups (**Supplementary Figures S7G, H**). Peroxiredoxin 4 (*Prdx4*), a new favorable regulator that protects oocytes against oxidative damage, showed no significant changes in the stress group (**Supplementary Figure S7I**). Gamma-histone *H2ax*, which indicates DNA breakage, also showed similar expression levels between the groups (**Supplementary Figure S7J**). NADH: ubiquinone oxidoreductase subunit B10 (*Ndufb10*), which is involved in mitochondrial activation, was unaffected by chronic stress (**Supplementary Figure S7K**).

Collectively, these results demonstrate that multiple hits on cAMP signaling pathways under chronic stress conditions are most likely responsible for MAF, which results in accelerated oocyte loss and premature ovarian aging.

## Discussion

Psychological well-being is closely related to female fertility (29). Female infertility can induce a highly stressed psychological state. However, the effect of stress on fertility remains unclear. The ovarian reserve is critical for female fertility. Psychological stress has been reported to be associated with diminished ovarian reserves in humans (2). How stress influences the ovarian reserve also needs to be elucidated.

In our study, we constructed a chronic stress mouse model to study the influence of mild chronic stress on the ovarian reserve. We chose a mild stress procedure in our study to mimic social stress conditions in humans. We observed a lag in body weight changes and a mild elongation of the estrus cycle in the stress group. These results indicated the successful construction of the stress model in our study. However, serum hormones showed no significant change after stress, which was a mild phenotype compared to previous studies (10). This difference might be due to the shorter stressing time in our study and differences in mouse species used in different studies.

The ovarian reserve is mainly composed of thousands of primordial follicles. However, in previous studies, no more than 200 primordial follicles could be calculated in each ovary using tissue slicing (10). In contrast, large follicles may easily be counted repeatedly because they might be cut into multiple sections on several slices. For example, there were approximately 100 corpus luteum per ovary based on tissue slicing in previous reports, which was much higher than the actual common level (10). Other studies have recommended that the numbers of primordial, primary, secondary, antral, and atretic follicles be counted in every 10th section, and the total be multiplied by 10 to give the estimated number of follicles in the whole ovary (8). We also calculated the number of primordial follicles in tissue slices. However, we found that primordial and primary follicles were not homogeneously distributed in the slices. Therefore, the method of multiplying has the risk of inducing false-positive or false-negative results. Under these conditions, significant changes in serum hormones and estrus cycles are critical for phenotypic analyses. Therefore, the influence of chronic stress on the ovarian reserve is largely uncertain, based only on follicle counting in tissue slices and hormone analyses.

Although no significant differences were detected in serum hormone levels, we observed a significant decline in the ovarian reserve in the stress group. To verify the influence of chronic stress on the ovarian reserve, we applied the established CUBIC method to reconstruct 3D ovarian fluorescence images at the single oocyte level (19, 21, 30–33). Oocytes from primordial follicles, clearly observed in the cortex of the 3D ovarian images, manifested very similar cell sizes. Statistical analysis revealed that both growing and quiescent oocytes were significantly decreased in the stress group. Compared with the results of tissue slicing in this study, which showed no obvious change in follicle number, the statistical results based on ovarian 3D images were more accurate for phenotypic analysis.

Oocyte loss is typically caused by follicular atresia. However, what occurs within the follicles, which leads to oocyte death and granulosa cell dysfunction, remains unclear. In our mouse model, a unique MAF phenotype was observed in the stress group. The follicles were at an early stage of follicle atresia with intact follicle morphology. However, the oocytes manifested precocious meiotic resumption at primary or secondary developmental stages. We could clearly view condensed chromosomes, assembled spindles, and extruded polar bodies, which are not observed in healthy immature follicles. These phenotypes were also associated with granular cell–oocyte gap-junction breakdown, which is crucial for maintaining oocyte meiotic arrest. However, it is challenging to quantify the MAF oocytes in an intact ovary by CUBIC method. Proper antibodies indicating MAF were critical in MAF identifying. In this study, whole-mount analysis of MAF oocyte number was not conducted.

When exploring the potential molecular mechanisms underlying MAF that resulted in follicle atresia and oocyte degeneration, we detected a significant downregulation of *Gpr3* but not of *Pde3a* in the stress group (**Supplementary Figure S7A**). This result indicates that the reduced synthesis efficiency of cAMP, but not the increased hydrolysis, might be responsible for MAF. We also found that stress induced the downregulation of *Nppc*, *Npr2*, and *Gja4*, which regulate the synthesis of cGMP in cumulus cells and are responsible for the transfer of cGMP to the oocytes to inhibit the hydrolysis of cAMP (**Figures 4G–I**). This result was in agreement with the histological phenotype of the gap-junction breakdown, which showed denuded oocytes in antral follicles and deciduous cumulus cells in the antral follicles. We propose that multiple hits on the cAMP signaling pathway contribute to MAF under stress conditions. However, the mediators within the ovary that are activated by stress and participate in the regulation of *Gpr3*, *Nppc*, *Npr2*, and *Gja4* remain unknown. Whether stress hormones directly regulate the expression levels of these genes requires further verification.

Our results revealed that chronic stress is detrimental to the ovarian reserve in mice. We also provided an approach for accurate ovarian reserve analysis using CUBIC at the single-oocyte level. In addition, we propose that multiple hits on the cAMP signaling pathways are likely to be critical mechanisms underlying stress-induced oocyte loss, providing potential molecular targets for the treatment of ovarian aging.

## Data availability statement

The original contributions presented in the study are included in the article/**Supplementary Material**. Further inquiries can be directed to the corresponding authors.

## Ethics statement

The animal study was approved by Institutional Review Board of Fudan University. The study was conducted in accordance with the local legislation and institutional requirements.

## Author contributions

Conceptualization: LZ, YJ, YK, and CX. Conduct of experiments and data generation: YJ, LZ, JX, CT, YL, XL, KL, QL, and HS. Analysis of data and interpretation: YJ, LZ, JX, CT, SH, GY, and YL. Manuscript preparation and editing: YJ, YS, FZ, YK, CX, and LZ. All authors contributed to the article and approved the submitted version.
