# Supplementary material for: Chronic stress induces meiotic arrest failure and ovarian reserve decline via the cAMP signaling pathway
Source: Front Endocrinol (Lausanne). 2023 Aug 30;14:1177061. doi: 10.3389/fendo.2023.1177061 (PMC10499613; doi:10.3389/fendo.2023.1177061)
Supplement: Supplementary file 1 [file DataSheet_1.docx]

**Supplemental tables**

Table S1. ELISA kit used in serum hormone detection.

| Hormone | Range | Company | Cat. No |
| --- | --- | --- | --- |
| AMH | 0.313 - 20 ng/ml | LSBio | LS-F22218 |
| FSH | 1.56 - 100 ng/ml | LSBio | LS-F38325 |
| LH | 0.47 - 30 ng/ml | LSBio | LS-F22503 |

Table S2. Reagents in CUBIC clearing method.

| Reagents | Company | Cat. NO |
| --- | --- | --- |
| PFA | Beyotime | P0099 |
| Sugar | Beyotime | ST1670 |
| Urea | Beyotime | ST1731 |
| Triton-X 100 | Sigma | X100 |
| Quadrol | Sigma | 122262 |
| Triethanolamine | Sigma | V900257 |

Table S3. Antibodies used in ovarian reconstruction analysis.

|  | Species | Company | Cat. No | Target |
| --- | --- | --- | --- | --- |
| **Primary antibodies** | | | | |
| DDX4 | Rabbit | Abcam | ab13840 | Oocyte cytoplasm |
| P63 | Mouse | Abcam | ab735 | Oocyte nucleus |
| **Secondary antibodies** | | | | |
| Alexa-647 | Donkey anti mouse | Invitrogen | A32787 |  |
| Cyanine3 | Goat anti rabbit | Invitrogen | A10520 |  |
| **Reagents** | | | | |
| DAPI |  | Sigma-Aldrich | D9542 |  |

Table S4. Primers used in mRNA expression analysis.

| Gene | Sequence of forward primer (5' to 3') | Sequence of forward primer (5' to 3') |
| --- | --- | --- |
| *Pde3a* | GAGGACCAAGGAAGAGATTC | CTCTCTTGTGGTCCCATTCT |
| *Nppc* | ATGCACCTCTCCCAGCTGAT | TCTCCCTTGAGATTGGCTCC |
| *Npr2* | TCACTACTTCACCATCGAGG | AGGATCTCATGCAGCATCTC |
| *Gja4* | GCACCAACGTCTGCTATGAC | AGACAGGTAGATGACGTGGC |
| *Gpr3* | CTCACCAGAGATGAGCTTGA | AAGGCCAGCATCACATAAGT |
| *Zfp36* | CGAGAGCCTCCAGTCGATGAG | GGATGGAGTCCGAGTTTATGTTCC |
| *Mapk1* | CTCAGCAATGACCACATCTG | ATCACAAGTGGTGTTCAGCA |
| *Elk1* | ATCCCTGCTCCCCACACATAC | CCACTGGACGGAAACTGGAA |
| *Egr1* | CCGAGCGAACAACCCTATGA | TGGGATAACTCGTCTCCACCAT |
| *Zp3* | AGGACTAACCGTGTGGAGGT | TCAGGCGAAGAGAGAAAGCC |
| *Gdf9* | CCCAAACCCAGCAGAAGTCA | AAACAGCAGGTCCACCATCG |
| *H2ax* | CGGTGGGCTTGAAGGTTAGT | ACTGGTATGAGGCCAGCAAC |
| *Prdx4* | CGAAGACAAGGAGGACTGGG | CGTCCACTGATCTTCCGACA |
| *Ndufb10* | CTCGCTCCCTAACCCCATCA | TTCTTGGCATGCTGTCGTTC |

**Supplemental Figures**

**
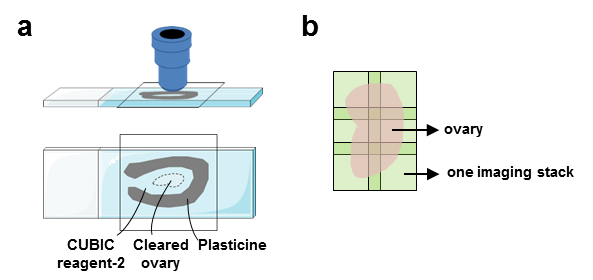
**

**Figure S1. Diagrammatic images of mounting and imaging on microscopy.** (a) Butyl rubber was used to make a U-shaped groove on the slice. CUBIC-reagent 2 was full fill the groove and ovaries were mounted within it. (b) Each ovary required 2×2 or 3×2 stacks of imaging area. 10% overlap was need for adjacent stacks.

**
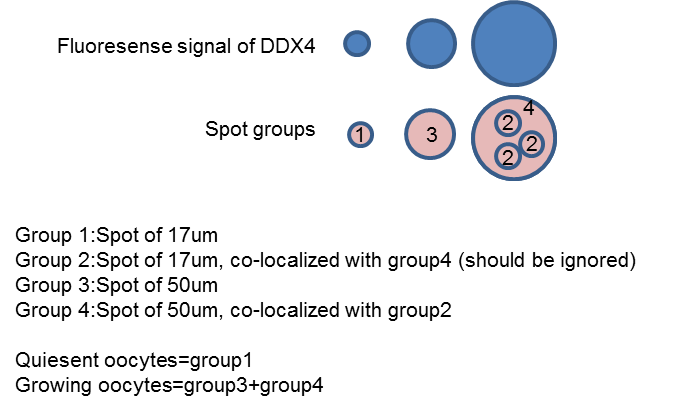
**

**Figure S2. Diagrammatic images of spot identification in Imaris software.** 4 groups of spots were gained by setting 17 um and 50 um as cell size parameters when construction spot groups and analyzing the co-localization. Large oocytes could be re-recognized by small spots. Therefore, “co-localize” algorism was used to distinguish group 2 from group 1 and group 4.

**
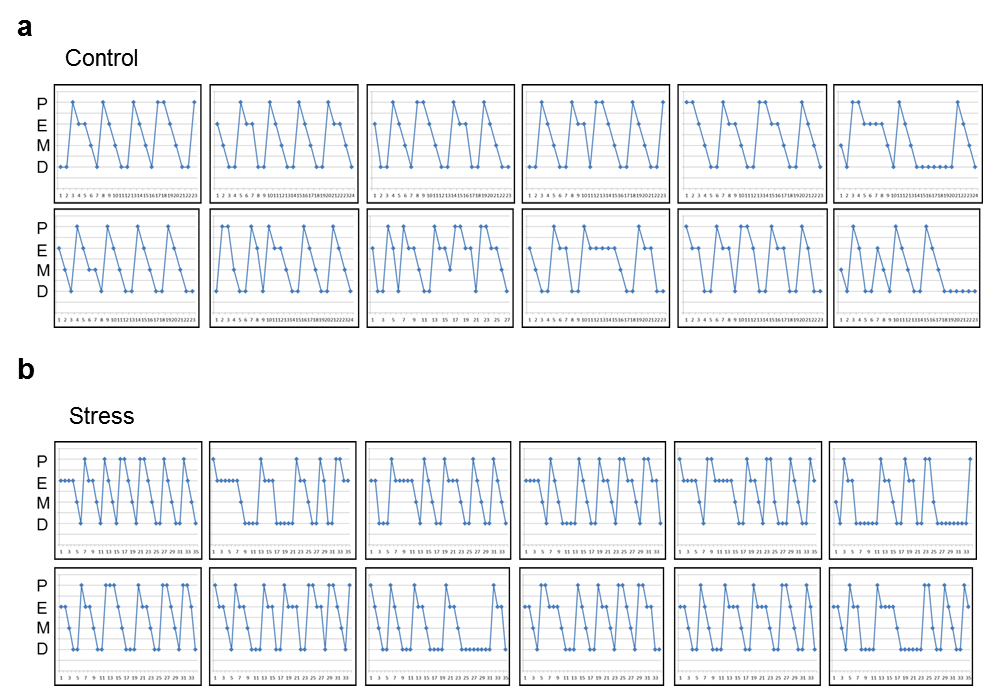
**

**Figure S3 Estrus cycle analysis in the control and stress group.** Estrus stage analysis in the control group (a) and the stress group (b). 12 mice were included in each group. Vaginal cells were collected by saline washes and analyzed by Wright-Giemsa staining. P, proestrus; E, estrus; M, metestrus; D, diestrus.


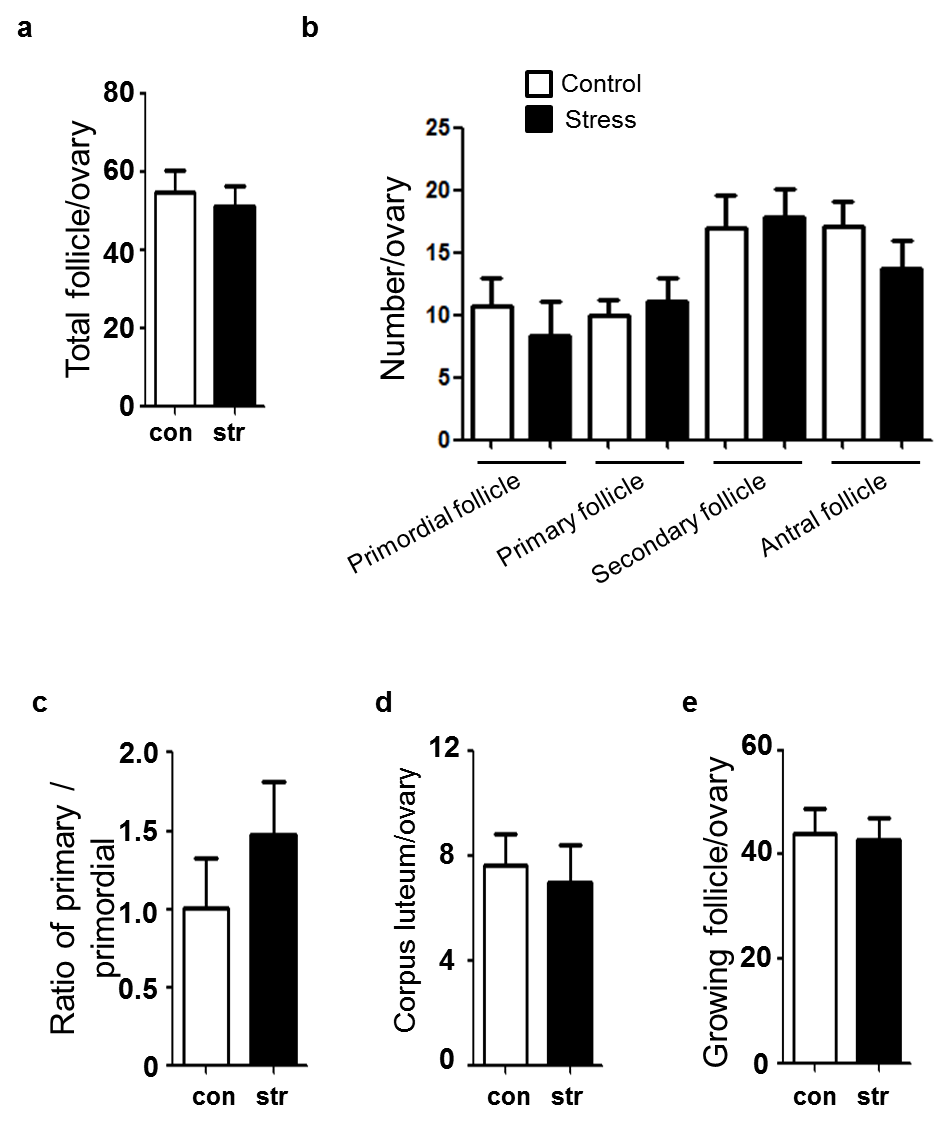


Figure S4. Follicle developmental analysis under tissue slicing and H&E staining. 11 ovaries and 7 ovaries were used in the control group and the stress group respectively for serial slicing and H&E staining. 70-90 slices could be gained for each ovary. The middle slice from every 30 slices were used for light microscopy and follicle analysis. Therefore, three slices were selected for follicle quantification for each ovary and the total number of follicles were used to indicate the follicle number of each ovary. (a) No significant difference of total follicle number was observed between groups. (b) No significant difference of primordial follicle, primary follicle, secondary follicle, antral follicle number was detected between groups. (c) No significant difference of the normalized ratio of primary follicle number to primordial follicle number was detected between groups. (d) No significant difference of corpus luteum number was detected between groups. (e) No significant difference of growing follicle number was detected between groups.


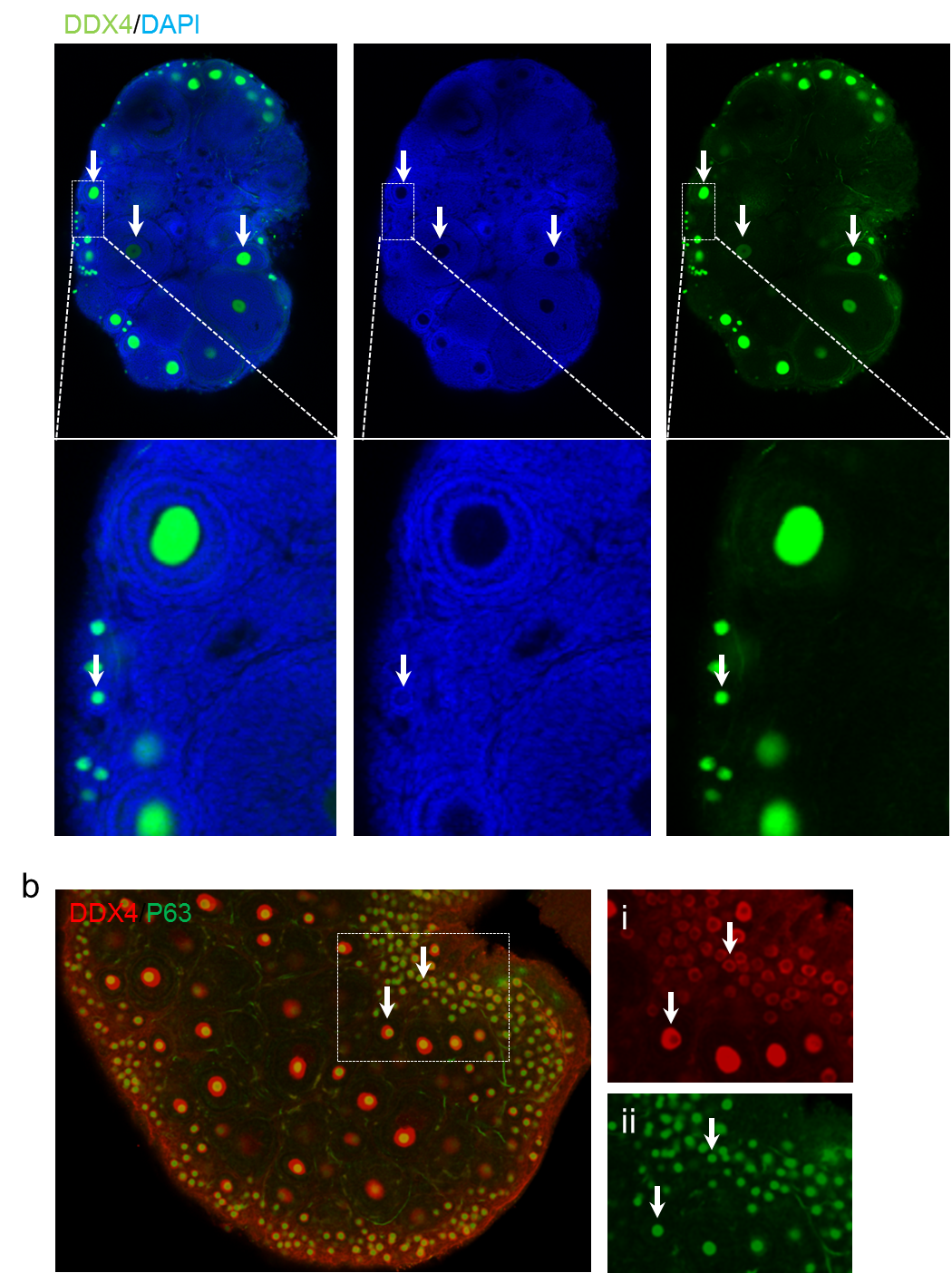


Figure S5. Antibody specificity test.(a) Ovarian tissue slices were stained by DDX4 (ab13840, Abcam) and DAPI (D9542, Sigma-Aldrich). DDX4 was germ-cell specific marker located in the cytoplasm of oocyte. Goat anti-rabbit (Alexa-cy3) fluorescence antibody was used (green).Cell nucleus were indicated as blue. White arrows indicted the oocytes from growing follicles in the upper row and quiescent oocyte from primordial follicles in the row below. (b) Double staining of DDX4 and P63 on rat ovarian slices. P63 is germ cell specific marker expressed in the nucleus. Fluorescence antibodies corresponding to DDX4 (ab13840, Abcam) and P63 (ab735, Abcam) included goat anti-rabbit (Alexa-cy3) and donkey anti-mouse (Alexa-647) respectively. DDX4 positive signals (red) were co-localized within P63 positive cells (green). The arrows indicated the oocytes in growing follicles and primordial follicles.


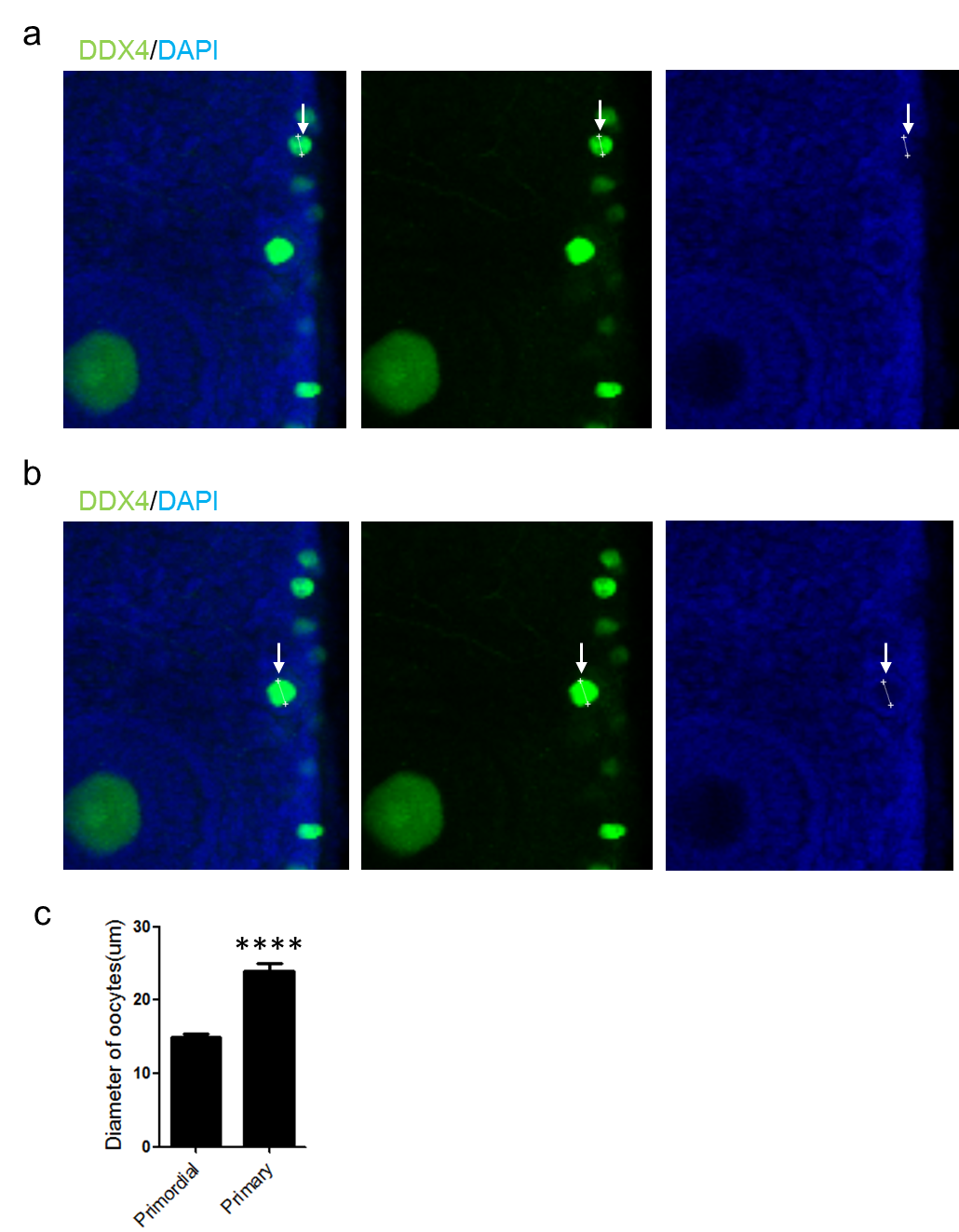


Figure S6. Classification of primordial oocytes and primary oocytes. (a) Primordial oocytes were in a large amount and distributed on the ovarian surface. (b) Primary oocytes were located relatively inner than primordial oocytes and surrounded by a layer of round granulosa cells. (c) Statistical analysis of cell size between primordial oocytes and primary oocytes. The average diameter of primordial oocytes was 15.0±0.3 μm (Mean±SEM，n=17) and this number became 23.9±1.1 μm (Mean±SEM，n=17) for primary oocytes. Arrows indicated the oocytes in measurement. ****, p<0.0001. t-test was used for different significance analysis.


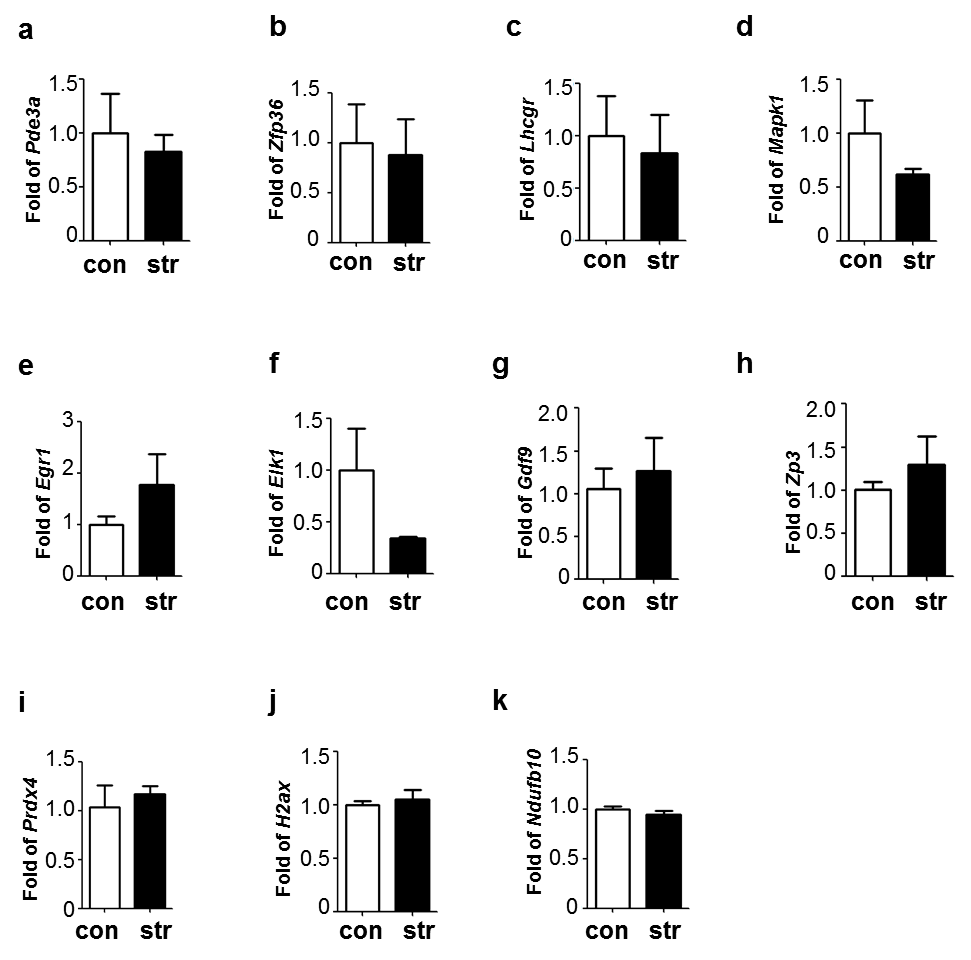


Figure S7. Expression level of genes in the control and the stress group. (a-k) Relative expression level of *Pde3a*, *Zfp36*, *Lhcgr*, *Mapk1*, *Egr1*, *Elk1*, *Gdf9*, *Zp3*, *Prdx4*, *H2ax* and *Ndufb10* to *Actin*. At least three repeats were included in each group. con, control; str, stress.
